# Si-Wei-Qing-Gan-Tang Improves Non-Alcoholic Steatohepatitis by Modulating the Nuclear Factor-κB Signal Pathway and Autophagy in Methionine and Choline Deficient Diet-Fed Rats

**DOI:** 10.3389/fphar.2020.00530

**Published:** 2020-05-01

**Authors:** Lingyun Xiao, Shu Liang, Lanlan Ge, Shuling Qiu, Haoqiang Wan, Shipin Wu, Jia Fei, Shusong Peng, Xiaobin Zeng

**Affiliations:** ^1^Centre Lab of Longhua Branch and Department of Infectious Disease, 2nd Clinical Medical College (Shenzhen People's Hospital) of Jinan University, Shenzhen, China; ^2^Integrated Chinese and Western Medicine Postdoctoral Research Station, Jinan University, Guangzhou, China; ^3^Department of Pathology (Longhua Branch), 2nd Clinical Medical College (Shenzhen People's Hospital) of Jinan University, Shenzhen, China; ^4^Guangdong Key Laboratory of Regional Immunity and Diseases, Shenzhen University School of Medicine, Shenzhen, China

**Keywords:** Si-Wei-Qing-Gan-Tang, non-alcoholic steatohepatitis, network pharmacology, inflammation, NF-κB, autophagy

## Abstract

Si-Wei-Qing-Gan-Tang (SWQGT) is a Chinese medicine formula that is widely used as a folk remedy of herbal tea for the treatment of chronic hepatitis, like non-alcoholic steatohepatitis (NASH), around Ganzhou City (Jiangxi province, China). However, the underlying mechanisms of this formula against NASH are still unknown. This study aimed to explore the effect and mechanisms of SWQGT against NASH. A network pharmacology approach was used to predict the potential mechanisms of SWQGT against NASH. Then a rat model of NASH established by feeding the methionine and choline deficient (MCD) diet was used to verify the effect and mechanisms of SWQGT on NASH *in vivo*. SWQGT (1 g/kg/d and 3 g/kg/d) were given by intragastric administration. Body weight, liver weight, serum biochemical indicators, liver triglyceride and total cholesterol were all measured. Tumor necrosis factor-α (TNF-α), Interleukin (IL)-1β, IL-6 levels in the livers were evaluated using ELISA. Hematoxylin and eosin (HE) and Oil Red O staining were used to determine histology, while western blot was used to assess the relative expression levels of the nuclear factor-κB (NF-κB) pathway- and autophagy-related proteins. Functional and pathway enrichment analyses revealed that SWQGT obviously influenced inflammation-related signal pathways in NASH. Furthermore, *in vivo* experiment showed that SWQGT caused a reduction in liver weight and liver index of MCD diet-fed rats. The formula also helped to reduce hepatomegaly and improve pathological liver changes and hepatic steatosis. SWQGT likewise reduced liver TNF-α, IL-1β, and IL-6 levels and down-regulated p-NF-κB p65, p-p38 MAPK, p-MEK1/2, p-ERK1/2, p-mTOR, and p62, while up-regulating p-ULK1 and LC3II protein expression levels. SWQGT could improve NASH in MCD diet-fed rats, and this effect may be associated with its down-regulation of NF-κB and activation of autophagy.

## Introduction

Non-alcoholic fatty liver disease (NAFLD) is a metabolic liver disease characterized by excess lipid deposition in hepatocytes, and its disease spectrum includes non-alcoholic steatohepatitis (NASH), fibrosis, cirrhosis, and hepatocellular carcinoma (HCC) (Ullah et al., 2019). The current global incidence of NAFLD is about 25.24%, and approximately 59% of the NALFD patients with structural liver biopsy have progressed to NASH (Younossi et al., 2016). Remarkably, NASH has become the second most common cause for liver transplantation, while NAFLD and NASH are the second major cause of HCC (Fazel et al., 2016).

The pathogenesis of NASH is not fully understood. Current theories suggest that inflammation is an important factor in the development of simple steatosis to NASH (Pierantonelli and Svegliati-Baroni, 2019). Inflammation not only causes liver cell injury and apoptosis, but also promotes liver lipid accumulation (Kim and Lee, 2018). In NAFLD, signals derived from fat tissues and intestinal bacteria flora, promote the release of inflammatory cytokines, and cause inflammation in the liver (Bessone et al., 2019). Nuclear factor-κB (NF-κB) transcription factor stimulated by various factors is linked closely to metabolism and plays a key role in inflammation (Cai et al., 2018). NF-κB activation increases the production of downstream inflammatory factors, such as Tumor necrosis factor-α (TNF-α), Interleukin (IL)-1β, and these inflammatory factors have been reported to enable steatosis and liver tissue damage, promoting the occurrence and development of NASH (Watanabe et al., 2004; Kubes and Mehal, 2012). Recent studies have shown that autophagy also plays a significant role in the occurrence and development of NASH. Its activation has been reported to reduce intracellular lipid droplets and attenuate inflammation, thus the regulation of autophagy may be a potential therapeutic approach in NAFLD/NASH (Allaire et al., 2019).

There are currently no agents approved for the treatment of NASH (Konerman et al., 2018). Thus, there is a need for safe and effective NASH drugs pending in-depth investigations. Various Chinese herbs have shown varying degrees of improvement in NASH and are predominantly safe (Bagherniya et al., 2018). Chinese medicine formula is a combination of several Chinese herbal medicines with multi-target and multi-mechanism features, thus it is plausible that these substances could provide a potential healing impact on the multifactorial NASH (Jadeja et al., 2014; Fiorucci et al., 2018). Some traditional Chinese medicine (TCM) formulae, such as the ErChen and QuYuHuaTanTongLuo Decoctions, have been proved to have a certain degree of therapeutic effect on NASH patients (Zhang et al., 2008).

The main pathogenesis of NASH in the perspective of TCM is dampness-heat retention, and the corresponding treatment is clearing heat and removing dampness. Si-Wei-Qing-Gan-Tang (SWQGT) is a Chinese medicine formula that constitutes *Artemisia capillaris* Thunb, *Hedyotis diffusa* Willd, *Gardenia jasminoides* Ellis, and *Taxillus sutchuenensis* (Lecomte) Danser following the clearing heat and removing dampness principles. This formula has been prescribed widely as a folk remedy of herbal tea for improving the symptoms of chronic hepatitis, like NASH, in Ganzhou City, China. Moreover, active ingredients in these herbs, like Quercetin (Marcolin et al., 2013), have been reported to reduce liver fat and inflammation and alleviate liver damage, which points to a potential therapeutic effect on NAFLD/NASH. Despite the extensive use of SWQGT by folk healers, neither scientific experiments nor clinical trials have been carried out to verify its effectiveness or explore its underling mechanisms against NASH.

With rapid development of bioinformatics, network pharmacology provides a new method to predict or reveal the complex mechanisms of TCM formula (Zuo et al., 2018). In the present study, we performed a network pharmacology approach to predict the potential pathways of SWQGT. Then, a rat model of NASH was established by feeding the methionine and choline deficient (MCD) diet, and used to verify the effect and mechanisms of SWQGT on NASH *in vivo*. While animal experiments are the best way to predict human response (Garattini and Grignaschi, 2017), the results, we hope, will provide a basis for further clinical research of SWQGT.

## Materials and Methods

### Chemicals and Reagents

The components of SWQGT are shown in **Table 1**, with all four herbs collected from Ganzhou City (Jiangxi province, China) in July 2018. The plants were identified by Dr. X.B.Z. of the Shenzhen People's Hospital, and voucher specimens (no. 2018091301, 2018091302, 2018091303, and 2018091304 for *A. capillaris*, *H. diffusa*, *G. jasminoides*, and *T. sutchuenensis*, respectively) were deposited at Center Lab of Longhua Branch, Shenzhen People's Hospital, Second Clinical Medical College of Jinan University, Shenzhen, China. Sprague Dawley (SD) rats (License Number: SCXK (Lu) 20140007) were purchased from Jinan Pengyue Laboratory Animal Breeding Co., Ltd (Jinan province, China). The MCD diet and methionine and choline-sufficient (MCS) diet were processed by Trophic Animal Feed High-Tech Co., Ltd (Nantong City, Jiangsu province, China). The triglyceride (TG) assay kit, total cholesterol (TC) assay kit, high-density lipoprotein cholesterol (HDL-c) assay kit, low-density lipoprotein cholesterol (LDL-c) assay kit, alanine aminotransferase (ALT) assay kit, aspartate transaminase (AST) assay kit, Oil Red O stain kit, and myeloperoxidase (MPO) assay kit were obtained from the Nanjing Jiancheng Bioengineering Institute (Nanjing City, Jiangsu province, China). TNF-α, IL-1β, and IL-6 ELISA kits were acquired from Shanghai Jianglai Biotechnology Co., Ltd (Shanghai City, China). Nuclear protein extraction kit was from Shanghai Sangon Biotech Co. Ltd (Shanghai City, China). TRIzol™ Reagent was obtained from Invitrogen (Carlsbad, CA, USA). RevertAid First Strand cDNA Synthesis Kit was from Thermo Fisher Scientific Inc (Waltham, MA, USA). Forget-Me-Not™ EvaGreen^®^ qPCR Master Mix was obtained from Biotium (Hayward, California, USA). Antibodies against NF-κB p65, p-NF-κB p65 (Ser536), p38 MAPK, p-p38 MAPK (Thr180/Tyr182), MEK1/2, p-MEK1/2 (Ser217/221), ERK1/2, p-ERK1/2 (Thr202/Tyr204), PI3K, AKT, p-AKT (Ser473), mTOR, p-mTOR (Ser2481), ULK1, p-ULK1 (Ser555), LC3A/B, GAPDH, and Lamin B1 were obtained from Cell Signaling Technology (Danvers, MA, USA). Anti-p62 antibody was from Beyotime Institute of Biotechnology (Shanghai City, China). The hepatoprotective drug Polyene Phosphatidylcholine Capsule (PPC, Essentiale, Sanofi-aventis Pharma Ltd.) was used as a positive functional control (He et al., 2017).

**Table 1:** Components of SWQGT.

Chinese name		Botanical name	Part used	Proportion
Yinchen	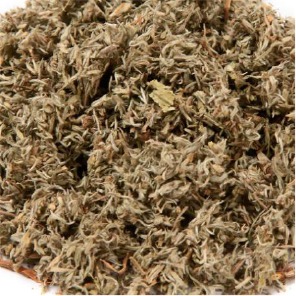	*Artemisia capillaris* Thunb	Aerial part	1
Baihuasheshecao	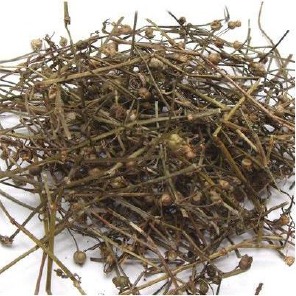	*Hedyotis diffusa* Willd	Herb	1
Zhizi	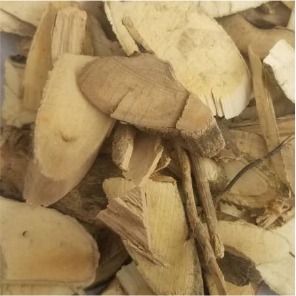	*Gardenia jasminoides* Ellis	Root	0.5
Sangjisheng	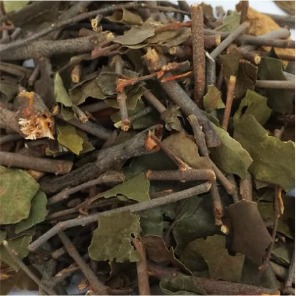	*Taxillus sutchuenensis (Lecomte)* Danser	Stem and leaf	0.5

### Preparation of SWQGT

SWQGT was boiled twice for 1 h each in 300 ml of water. The aqueous extracts were mixed and concentrated to 3 g/ml (crude herbal concentration), then filtered through a 0.22 μm microporous membrane, with the resulting solution ready for use. Identification of major compounds in the herbs of SWQGT for quality control was conducted using ultra-performance liquid chromatography-quadrupole time of flight mass spectrometry (UPLC-QTOF-MS) system, equipped with a UPLC apparatus (Ultimate 3000, Thermo Fisher Scientific, USA) and a QTOF-MS mass analyzer (Maxis Impact, Bruker, Germany). The chromatographic separation was performed on an Agilent Eclipse Plus C_18_ column (50 mm, 2.1 mm ID, 1.8 μm). The aqueous phase was a mixture of acetonitrile (A) and water (B), and the gradient elution procedure was set as follows: 0–20min, 5%–13% A; 20–50 min, 13–40% A; 50–60 min, 40–80% A. The mass analyses were performed using an ESI interface in the negative ion mode with the following operation parameters: capillary voltage 4500 V; end plate offset, −500 V; nebulizer pressure, 0.4 bar; drying gas, 6 L/min and gas temperature 180˚C. Full scan mass spectra were recorded over the range 50–1500 m/z. The UPLC chromatograms of SWQGT and its single herb were shown in **Figure S1**. The results of UPLC-QTOF-MS and tentative identification by comparison to reports from literature were shown in **Table S1**.

### Prediction of the Mechanisms of SWQGT Against NASH Based on Network Pharmacology

The ingredients of four herbs in SWQGT were retrieved from Traditional Chinese Medicines Systems Pharmacology (TCMSP, http://tcmspw.com/tcmsp.php) (Ru et al., 2014). Evaluation of the ADME (Absorption, Distribution, Metabolism and Excretion) was used to predict the pharmacokinetics of the components. Ingredients with OB ≥ 30% and DL ≥ 0.18 were chosen for further analyses (Xu et al., 2012). The protein targets of these components were retrieved from TCMSP and DrugBank databases, and standardized using UniProt KB database (Ru et al., 2014; Lee, 2015). The list of NASH-related targets were collected from OMIM (https://omim.org/) and DisGeNET (https://www.disgenet.org/) using the search term of “nonalcoholic steatohepatitis” and “nonalcoholic fatty liver disease” (Hamosh et al., 2005; Pinero et al., 2017). To predict the mechanisms of SWQGT against NASH, a Component-Target network was constructed with overlapped targets, and plotted using Cytoscape (Cai et al., 2019). Putative targets were further put into DAVID (https://david.ncifcrf.gov/) for enrichment analyses in KEGG (Kyoto Encyclopedia of Genes and Genomes) pathways and GO (Gene Ontology) terms associated with biological processes (BP), molecular functions (MF), and cellular components (CC).

### Animal Experiments

SD rats (male, 7 weeks old, 201 ± 10.7 g, mean ± SD) were kept at the SPF animal center (12 h of daylight cycle, 18˚C–22˚C). After acclimation for 2 weeks, the rats were assigned to five groups with a table of random numbers (eight rats per group): the MCS group, MCD group, SW-L group (low dose SWQGT group), SW-H group (high dose SWQGT group), and PPC group. Briefly, random numbers between 0 and 1 generated using the RAND() command in EXCEL (Microsoft Corporation, USA) were assigned to each rat. The random numbers were then sorted from small to large and assigned to groups of five in turn. Rats in the MCS group were fed with the MCS diet, while rats in the other four groups had the MCD diet. Additionally, rats in the SW-L group and SW-H group received SWQGT at a dose of 1 g/kg/d and 3 g/kg/d, respectively, *via* gavage, whereas rats in the MCS group and MCD group received equal amounts of water *via* gavage. PPC (120 mg/kg/d) was also given by intragastric administration. One rat in each group was randomly chosen to verify the pathological progress of NASH at 3 weeks, and these animals were excluded from the experiment. The other rats (n = 7 per group) were anesthetized with 10% chloral hydrate (3 ml/kg) at 4 weeks. Abdominal aortic blood was collected, and the rats were sacrificed. Following sacrifice, liver tissues of rats were harvested and weighed, and the liver indices were counted (liver index = liver weight/body weight × 100).

All animal treatments complied with the protocols approved by the Institutional Animal Care and Use Committee of the Shenzhen People's Hospital. The same committee approved this research (Approval Document No. LL-KT-201701017).

### Histology Determination

Liver tissues were cut into small pieces, fixed in neutral formalin for 24 h, dehydrated, embedded in paraffin, and then cut into 10 μm slices and stained with hematoxylin and eosin (HE). Liver tissue used for Oil Red O staining were frozen and sectioned into 10 μm slices, then stained with Oil Red O and counter-stained with hematoxylin.

### Biochemical Analyses

Abdominal aorta blood was centrifuged at 3,000 rpm for 10 min, after which the resulting serum was collected for further use. Nine hundred microliters of the homogenate medium was added to 100 mg of liver tissues, and the mixture was homogenized. The ensuing supernatant was collected after centrifugation at 3,000 rpm for 10 min. Serum TC, serum TG, serum HDL-c, serum LDL-c, serum AST, serum ALT, liver TG, and liver TC were evaluated according to the manufacturer's instructions. Liver TNF-α, IL-1β, and IL-6 were assessed following instructions for the procedure provided with the ELISA kits. The hepatic MPO activity was determined using an assay kit according to the manufacturer's instructions.

### Western Blotting

Protein expression levels were detected using western blot with antibodies of NF-κB p65 (1:1,000), p-NF-κB p65 (1:1,000), p38 MAPK (1:1,000), p-p38 MAPK (1:1,000), MEK1/2 (1:1,000), p-MEK1/2 (1:1,000), ERK1/2 (1:1,000), p-ERK1/2 (1:1,000), PI3K (1:1,000), AKT (1:1,000), p-AKT (1:2,000), TLR4 (1:1,000), mTOR (1:1,000), p-mTOR (1:1,000), ULK1 (1:1,000), p-ULK1 (1:1,000), LC3 (1:1,000), p62 (1:2,000), GAPDH (1:1,000), and Lamin B1 (1:1,000). Liver tissues were homogenized on ice in RIPA Lysis Buffer with protease inhibitors, and the supernatant was collected after centrifugation. A BCA Protein Assay Kit was then used for the assessment of protein concentration in the supernatant. For nuclear protein preparation, liver samples were extracted by a commercial kit according to the manufacturer's instructions. GAPDH was used as total protein loading controls, while Lamin B1 as a nuclear protein loading control. The samples were separated by SDS-PAGE gel and transferred to PVDF membranes. After blocking with 5% non-fat milk for 1 h at room temperature, the membranes were probed with the respective primary antibodies, followed by incubation with peroxidase-conjugated secondary antibodies. An ECL reagent was added to the membranes, and a chemiluminescent imaging system was used to visualize target proteins.

### Quantitative Real-Time PCR Analysis

MCP-1, EMR1 mRNA expression levels were determined by quantitative real-time PCR. Briefly, total RNA was isolated from the liver samples using TRIzol Reagent according to the manufacturer's protocol. Then, cDNA was synthesized by reverse transcription with a RevertAid First Strand cDNA Synthesis Kit. Quantitative real-time PCR was performed using Forget-Me-Not™ EvaGreen^®^ qPCR Master Mix. The fold change of gene expression was calculated through relative quantification (2^−ΔΔCt^). Primers used for qPCR are listed as follows: MCP-1 forward primer: TAGCATCCACGTGCTGTCTC; MCP-1 reverse primer: GAGCTTGGTGACAAATACTACAGC; EMR1 forward primer: TCTCTCTGGTATGTCTCGCCT; EMR1 forward primer: CGCAAGCTGTCTGGTTGTC; GAPDH forward primer: TGATGGGTGTGAACCACGAG; GAPDH reverse primer: TCATGAGCCCTTCCACGATG.

### Statistical Analyses

GraphPad Prism 7.0 were used for statistical analyses. Datasets of qPCR and western blot were analyzed using One-way ANOVA with Dunnett's multiple comparisons test, and these data are presented as mean ± standard deviation (SD). Other data were analyzed using Mann-Whitney U test, and these data are presented as median and interquartile range. *p* < 0.05 was considered to be statistically significant.

## Results

### Network Pharmacology Analyses of SWQGT

**Figure 1:**
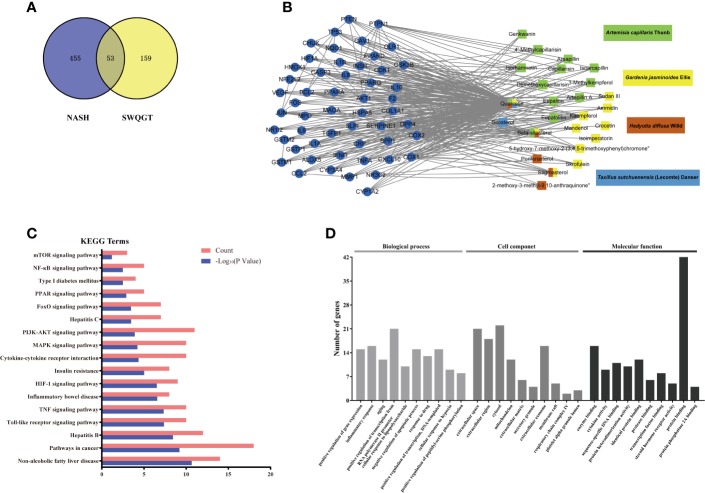
Network construction and function analyses of Si-Wei-Qing-Gan-Tang (SWQGT). **(A)** Venn diagram of candidate targets in SWQGT and non-alcoholic steatohepatitis (NASH). **(B)** The Component-Target network. **(C)** The KEGG pathway enrichment analyses of 53 putative targets. **(D)** The GO enrichment analyses of 53 putative targets.

In order to predict the potential mechanisms of SWQGT, we first perform a network pharmacology approach. Components of each herb in SWQGT were collected and 26 components were screened out with OB ≥ 30% and DL ≥ 0.18. Several of the selected components were identified as main constituents of SWQGT in **Table S1** using the UPLC-QTOF-MS system. A total of 212 potential targets of these components were predicted through database retrieval. Detailed information of components in SWQGT and their candidate targets were shown in **Table S2**. Comparing the candidate targets of SWQGT with 508 candidate targets relating to NAFLD/NASH from database there was an overlap of 53 targets, which were assumed as the putative targets of SWQGT against NAFLD/NASH and used to construct a Component-Target network (**Figures 1A, B**).

As predicted pathways by the bioinformatics methods showed higher consistency with microarray confirmation than predicted genes (Fang et al., 2017), we payed more attention to enriched pathways than specific genes. KEGG pathways in which the 53 putative targets showed significant enrichment contained toll-like receptor signaling pathway, TNF signaling pathway, cytokine-cytokine receptor interaction, NF-κB signaling pathway, etc, indicating that the effect of SWQGT against NAFLD/NASH was closely related to inflammation (**Figure 1C**). GO enrichment in terms associated with biological processes also revealed that SWQGT may regulate inflammatory response (**Figure 1D**).

### SWQGT Reduced Liver Weight and Liver Index in MCD Diet-Fed Rats

**Figure 2:**
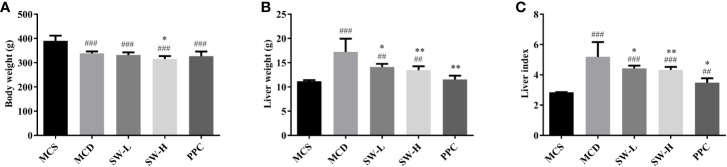
Effect of Si-Wei-Qing-Gan-Tang (SWQGT) and PPC on body weight, liver weight and liver index of rats in each group. **(A)** body weight, **(B)** liver weight, **(C)** liver index. Methionine and choline-sufficient (MCS) group: MCS diet-fed rats, methionine and choline deficient (MCD) group: MCD diet-fed rats, SW-L group: rats fed with low dose of SWQGT and MCD diet, SW-H group: rats fed with high dose of SWQGT and MCD diet, PPC group: rats fed with PPC and MCD diet. The data are presented as median and interquartile range, ^##^*p* < 0.01, ^###^*p* < 0.001, *vs.* MCS group; ^*^p < 0.05, ^**^p < 0.01 *vs.* MCD group.

To verify the effect of SWQGT on NASH, the MCD model in rats was used in our study. Compared with the MCS diet-fed rats, feeding on the MCD diet for 4 weeks caused body weight decrease (*p* < 0.001) (**Figure 2A**). Rats in SW-H group showed slight but significant weight loss when compared with the MCD group. The liver weight and liver index of rats in the MCD group were significantly higher than those of rats in the MCS group (*p* < 0.001). Rats in the PPC group showed significant reduced liver weight (*p* < 0.01) and liver index (*p* < 0.05), compared with rats in the MCD group (**Figures 2B, C**). Rats in the SW-L and SW-H groups also had lower liver weight and liver index than rats in the MCD group (*p* < 0.05, *p* < 0.01) (**Figures 2B, C**). Per these results, SWQGT reduced the liver weight and liver index gain caused by the MCD diet in rats.

### SWQGT Affected Serum Biochemical Indicators in MCD Diet-Fed Rats

**Figure 3:**
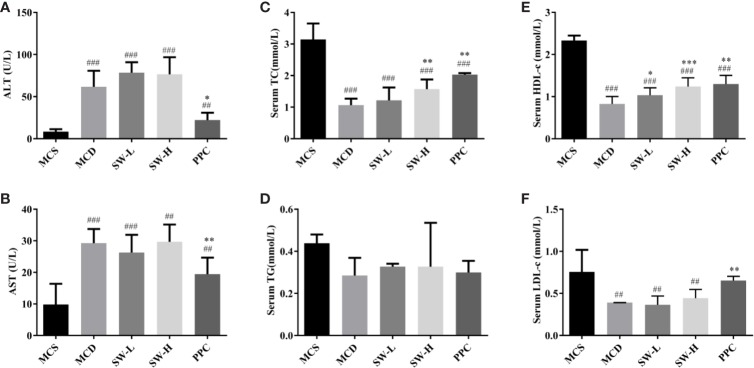
Effect of Si-Wei-Qing-Gan-Tang (SWQGT) and PPC on serum ALT, AST, total cholesterol (TC), triglyceride (TG), high-density lipoprotein cholesterol (HDL-c) assay kit, and low-density lipoprotein cholesterol (LDL-c) levels of rats in each group. **(A)** serum ALT, **(B)** serum AST, **(C)** serum TC, **(D)**: serum TG, **(E)** serum HDL-c, **(F)** serum LDL-c. Methionine and choline-sufficient (MCS) group: MCS diet-fed rats, methionine and choline deficient (MCD) group: MCD diet-fed rats, SW-L group: rats fed with low dose of SWQGT and MCD diet, SW-H group: rats fed with high dose of SWQGT and MCD diet, PPC group: rats fed with PPC and MCD diet. The data are presented as median and interquartile range, ^##^ *p* < 0.01, ^###^ *p* < 0.001 *vs.* MCS group; ^*^ *p* < 0.05, ^*^ *p* < 0.01, ^***^ *p* < 0.001 *vs.* MCD group.

The results of serum biochemical analyses were shown in **Figure 3**. The ALT and AST levels of rats in the MCD group were significantly higher than those in the MCS group (*p* < 0.001), and PPC treatment alleviated MCD diet-induced ALT and AST increase (*p* < 0.05, *p* < 0.01). However, there was no significant difference in the levels of these indicators between the SW-L and SW-H groups and the MCD group (**Figures 3A, B**). There was no significant difference in serum TG levels between the five groups (**Figure 3D**). Serum TC, HDL-c, and LDL-c levels in the MCD group were significantly lower than those in the MCS group (*p* < 0.001, *p* < 0.001, *p* < 0.01), while all three indicators in the PPC group were increased significantly when compared with the levels in the MCD group (*p* < 0.01) (**Figures 3C–F**). HDL-c levels in the SW-L and SW-H groups were significantly higher than those in the MCD group (*p* < 0.05, *p* < 0.001), and serum TC levels in the SW-H group were significantly higher those in the MCD group (*p* < 0.01) (**Figures 3C, E**).

### SWQGT Decreased Liver Fat Accumulation in MCD Diet-Fed Rats

**Figure 4:**
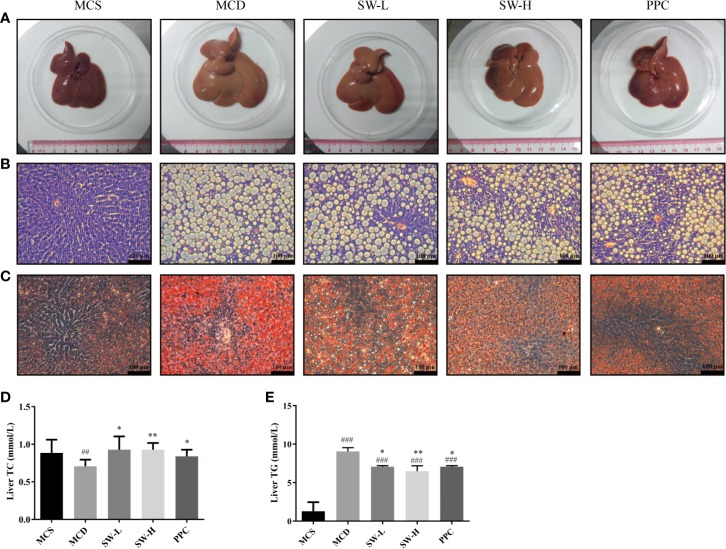
Effect of Si-Wei-Qing-Gan-Tang (SWQGT) and PPC on histopathological examination and liver total cholesterol (TC), TG levels of rats in each group. **(A)** liver morphology, **(B)** HE stained sections (× 200), **(C)** Oil Red O stained sections (× 200), **(D)** liver TC levels, **(E)** liver triglyceride (TG) levels. methionine and choline-sufficient (MCS) group: MCS diet-fed rats, methionine and choline deficient (MCD) group: MCD diet-fed rats, SW-L group: rats fed with low dose of SWQGT and MCD diet, SW-H group: rats fed with high dose of SWQGT and MCD diet, PPC group: rats fed with PPC and MCD diet. The data are presented as median and interquartile range, ^##^ *p* < 0.01, ^###^ *p* < 0.001 *vs.* MCS group; ^*^ *p* < 0.05, ^**^ *p* < 0.01 *vs.* MCD group.

The liver tissue morphology (**Figure 4A**) showed that the livers of rats in the MCS group were dark red, the capsules were normal, the edges were sharp, and the touch was elastic. In the MCD group, the livers of rats swelled, the color of the livers was yellowish, the films were tense, the edges were blunt, and the touch was softer. Rats in the SW-L, SW-H, and PPC groups, in terms of liver size, color, and touch, were better than those in the MCD group. HE staining results (**Figure 4B**) showed that the cell boundaries in the liver tissues of rats in the MCS group were apparent, the blue stained nucleus was at the center of the cell, the structure of the hepatic cord was clear, and there was no obvious lesion. A large number of fat vacuoles were found to exist in the liver tissues of the MCD group rats, and even adjacent cells fused into a piece. The cell boundaries of the tissues of rats in this group were unclear, the nucleus was squeezed to one side, and the structure of the hepatic cord was not lucid enough. The fat vacuoles in the SW-L, SW-H, and PPC groups diminished relatively, compared to the MCD group, and the hepatic cord structures were restored to some extent.

Oil Red O staining (**Figure 4C**) showed that there was a large amount of orange-red lipid droplets in the liver tissues of the MCD group rats. Lipid droplets in the SW-L, SW-H, and PPC groups reduced relatively compared with the MCD group. Our analyses of the TG content in liver tissues revealed that TG levels increased significantly in the livers of the MCD group rats, compared with the levels in the MCS group rats (*p* < 0.001), whereas, the liver TG levels in the SW-L, SW-H,and PPC groups decreased significantly, compared with those in the MCD group animals (*p* < 0.05, *p* < 0.01, *p* < 0.05) (**Figure 4E**). In addition, the MCD group rats had lower liver TC levels, compared with levels in the MCS group rats, while rats in the SW-L, SW-H, and PPC groups showed restored TC levels (*p* < 0.05, *p* < 0.01, *p* < 0.05), compared with the MCD group (**Figure 4D**). These results demonstrated that SWQGT alleviated abnormal liver lipid accumulation and pathological changes caused by the MCD diet in rats.

### SWQGT Decreased NF-κB Activation and Reduced Liver Tissue Inflammation in MCD Diet-Fed Rats

**Figure 5:**
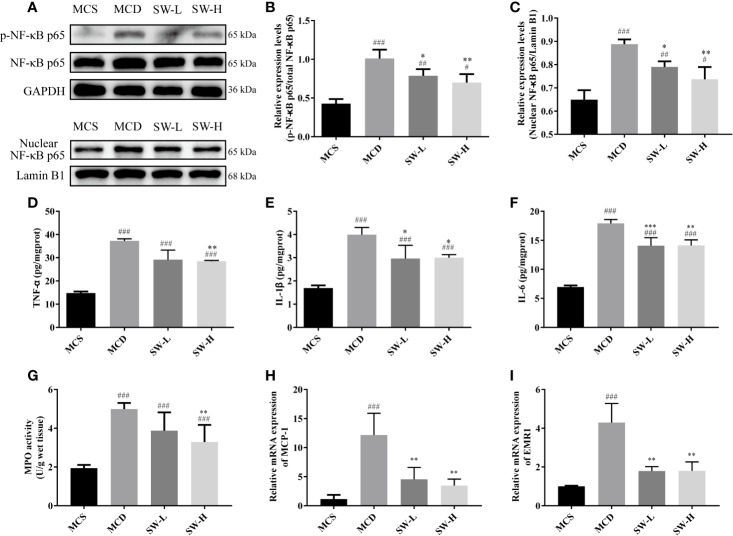
Effect of Si-Wei-Qing-Gan-Tang (SWQGT) on the nuclear factor-κB (NF-κB) protein and inflammatory mediators. **(A)** Western blot; **(B)** p-NF-κB p65 protein levels; **(C)** nuclear NF-κB p65 protein levels; **(D)** liver TNF-α levels; **(E)** liver IL-1β levels; **(F)** liver IL-6 levels; **(G)** liver MPO activity; **(H)** hepatic mRNA levels of MCP-1; **I**: hepatic mRNA levels of EMR1. Methionine and choline-sufficient (MCS) group: MCS diet-fed rats, methionine and choline deficient (MCD) group: MCD diet-fed rats, SW-L group: rats fed with low dose of SWQGT and MCD diet, SW-H group: rats fed with high dose of SWQGT and MCD diet. The data in **(B, C, H, I)** are presented as the mean ± SD, while data in **(D–G)** are presented as median and interquartile range. ^#^*p* < 0.05, ^##^*p* < 0.01, ^###^*p* < 0.001 *vs.* MCS group; ^*^p < 0.05, ^**^p < 0.01, ^***^p < 0.001 *vs.* MCD group.

Considering the enriched pathways of SWQGT in inflammation, as well as the vital role of NF-κB in the inflammatory response (Liu et al., 2017; Cai et al., 2018), we examined the expression of NF-κB-related proteins. The p-NF-κB p65 protein levels of liver tissues in the MCD group rats increased significantly compared with those in the MCS group (*p* < 0.001), whereas, the p-NF-κB p65 protein levels in the SW-L and SW-H groups decreased significantly, compared with those in the MCD group (*p* < 0.05, *p* < 0.01) (**Figures 5A, B**). In addition, nuclear levels of NF-κB p65 also increased significantly (*p* < 0.001), and they were significantly decreased in SW-L and SW-H groups when compared with those in the MCD group (*p* < 0.05, *p* < 0.01) (**Figures 5A, C**). These results suggest that NF-κB was activated in the livers of MCD diet-fed rats, and the intervention of SWQGT down-regulated the activation of NF-κB.

We also tested the levels of inflammatory factors related to NF-κB in the livers of rats of each group. Our findings show that rats in the MCD group had higher liver TNF-α, IL-1β, and IL-6 levels, compared with the MCS group rats (*p* < 0.001) and the SW-H group rats (*p* < 0.01, *p* < 0.05, *p* < 0.01) (**Figures 5D–F**). IL-1β and IL-6 levels were also decreased in the SW-L group when compared with those in the MCD group (*p* < 0.05, *p* < 0.001). Rats in the SW-H group also had decreased MPO activity (*p* < 0.01) compared with the MCD group rats (**Figure 5G**). mRNA expression of hepatic MCP-1 and EMR1 in both the SW-L and SW-H groups were lower than those in the MCD group (*p* < 0.01) (**Figures 5H, I**).

### SWQGT Decreased the MEK1/2/ERK1/2 and p38 MAPK Signal Pathways Activation

**Figure 6:**
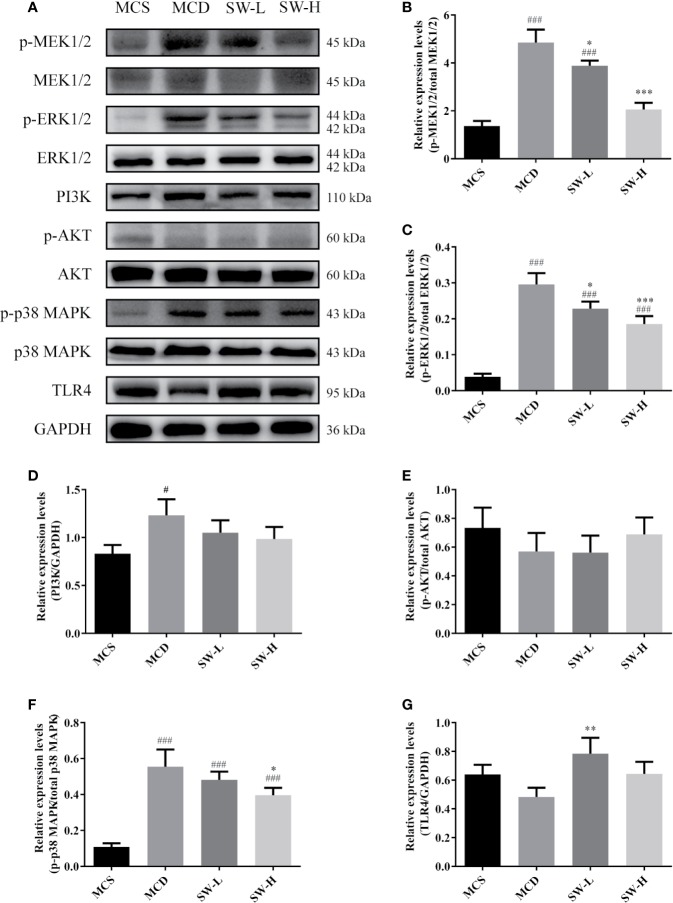
Effect of Si-Wei-Qing-Gan-Tang (SWQGT) on the levels of MEK/ERK, TAK1/p38 MAPK, and PI3K/Akt pathway related proteins. **(A)** Western blot; **(B)** p-MEK1/2 protein levels; **(C)** p-ERK1/2 protein levels; **(D)** PI3K protein levels **(E)** p-AKT protein levels; **(F)** p-p38 MAPK protein levels; **E** TLR4 protein levels. Methionine and choline-sufficient (MCS) group: MCS diet-fed rats, methionine and choline deficient (MCD) group: MCD diet-fed rats, SW-L group: rats fed with low dose of SWQGT and MCD diet, SW-H group: rats fed with high dose of SWQGT and MCD diet. The data are presented as the mean ± SD. ^#^*p* < 0.05, ^###^*p* < 0.001 *vs.* MCS group, ^*^p < 0.05, ^**^p < 0.01, ^***^p < 0.001 *vs.* MCD group.

We then examined the NF-κB upstream pathways, including the MEK1/2/ERK1/2, PI3K/Akt, p38 MAPK, and TLR4 signal pathways (Lee et al., 2017). The MAPK signaling pathway, PI3K-AKT signaling pathway, toll-like receptor signaling pathway were also included in the pathway enrichment results of SWQGT predicted by network pharmacology (**Figure 1C**). As shown in **Figures 6A–C**, the p-MEK1/2 and p-ERK1/2 protein levels in liver tissues of the MCD group rats were significantly higher than those of rats in the MCS group (*p* < 0.001) and SW-H and SW-L groups (*p* < 0.05, *p* < 0.001). The p-p38 MAPK protein levels in liver tissues of the MCD group rats were significantly higher than levels in the MCS group (*p* < 0.001) and SW-H group (*p* < 0.05) (**Figures 6A, F**). Likewise, the PI3K protein levels in liver tissues of the MCD group rats were significantly higher than those in the MCS group (*p* < 0.05), but there was no significant difference in the p-AKT and TLR4 protein levels between the MCD group and the MCS group (**Figures 6A, D, E, G**). The p-AKT protein levels in the SW-H and SW-L groups exhibited no significant differences relative to levels in the MCD group. Per these results, SWQGT down-regulated the activation of the MEK1/2/ERK1/2 and p38 MAPK pathways in the livers of MCD diet-fed rats.

### SWQGT Decreased mTOR and Regulated Autophagy-Associated Protein Levels

**Figure 7:**
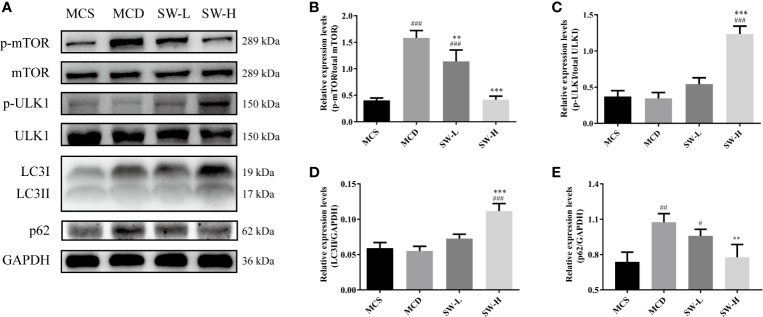
Effect of Si-Wei-Qing-Gan-Tang (SWQGT) on the levels of mTOR and autophagy related proteins. **(A)** Western blot; **(B)** p-mTOR protein levels; **(C)** p-ULK1 protein levels; **(D)** LC3II protein levels; **(E)** p62 protein levels. Methionine and choline-sufficient (MCS) diet-fed rats, methionine and choline deficient (MCD) group: MCD diet-fed rats, SW-L group: rats fed with low dose of SWQGT and MCD diet, SW-H group: rats fed with high dose of SWQGT and MCD diet. The data are presented as the mean ± SD. ^#^*p* < 0.05, ^##^*p* < 0.01, ^###^*p* < 0.001 *vs.* MCS group; ^**^p < 0.01, ^***^p < 0.001 *vs.* MCD group.

SWQGT showed a trend of enrichment in mTOR signaling pathway (*p* = 0.064) (**Figure 1C**). In addition, mTOR can be activated by the MEK1/2/ERK1/2 pathway, inhibiting autophagy (Kim et al., 2013). ULK1, a key protein for autophagy initiation, and LC3II, a marker of autophagosome formation, are potentially key to regulating autophagy (Wang et al., 2016). In our experiment, liver p-mTOR levels in the MCD group rats increased significantly (*p* < 0.001), compared with the animals in the MCS group (**Figures 7A, B**). p-ULK1 levels showed a decreasing trend with no significant difference between the MCS and MCD groups (**Figures 7A, C**). The SW-H group rats had lower levels of p-mTOR (*p* < 0.001), and higher levels of p-ULK1 and LC3II than those in the MCD group (*p* < 0.001, *p* < 0.001) (**Figure 7A–D**). In addition, protein expression of p62, an autophagic substrate usually used to evaluate the level of autophagy flux (Chen et al., 2016), was also reduced in the SW-H group compared with the MCD group (**Figure 7E**). These results showed that SWQGT inhibited mTOR and activated autophagy in the livers of MCD diet-fed rats.

## Discussion

The MCD diet is widely used in the studies of NASH, as it's modeling time is short, and the model is highly similar to NASH in histopathology (Li et al., 2018). The choline deficiency and the added methionine deficiency bring about a rapid onset of the NASH phenotype with lobular inflammation and ballooning (Li et al., 2018). However, the model's weight loss, low levels of blood lipids, and normal insulin sensitivity are not consistent with the clinical metabolic characteristics of NAFLD (Lau et al., 2018). This model is generally considered adequate to study the intrahepatic events in relation to NASH and the pharmacological treatment of NASH (Van Herck et al., 2017). As this study is the first to investigate the effect and mechanisms of SWQGT in liver diseases, we chose the MCD diet model. In our study, the rats developed NASH histological phenotypes after feeding the MCD diet for 4 weeks, which is consistent with previous reports (Van Herck et al., 2017). Remarkably, we observed in our investigation that SWQGT reduced liver weight and liver index in MCD diet-fed rats. Pathologic changes are key indicators for the outcomes of this model (Van Herck et al., 2017), and we found that SWQGT could improve MCD diet-induced morphological abnormality and hepatocyte ballooning. NASH patients are often accompanied by mild hepatomegaly (Tanaka et al., 2006), and the reduction of hepatic steatosis has also been associated with an improvement in hepatomegaly in another clinical study (Lin et al., 2017). SWQGT also induced a significant improvement in the liver steatosis of MCD diet-fed rats, backed by the TG levels of liver tissues, suggesting that SWQGT improved major indicators of NASH in this model. The effect of SWQGT on improving pathologic changes and liver steatosis was close to the positive control PPC, which is a clinically used hepatoprotective drug.

The AST and ALT levels are significantly increased after 4 weeks of MCD diet. However, we did not find, after 4 weeks of treatment with SWQGT, that the formula improved serum ALT and AST levels significantly. As liver biopsy remains the only standard for clinically diagnosing NAFLD/NASH (Bedossa, 2018), some investigations indicate changes in serum ALT and AST levels may be unreliable for the diagnosis and monitoring of the disease (Filozof et al., 2015). In addition, the serum ALT and AST levels are reported to have relatively large intra-individual variation (Botros and Sikaris, 2013). Several pharmacological studies in NASH also reported improved hepatic function with unaltered AST and/or ALT levels (Tahan et al., 2007; Kochi et al., 2014).

The metabolic context in MCD diet-fed animals is distinct from human NASH (Van Herck et al., 2017), therefore, this model is far from optimal to examine the metabolic parameters (Ibrahim et al., 2016). In our study, serum TG, TC, LDL-c, and HDL-c levels decreased in MCD diet-fed rats. The treatment of SWQGT alleviated the decline of serum TC and HDL-C, indicating that SWQGT might improve the overall health of MCD diet-fed rats. Unexpectedly, slight weight loss was seen in SW-H group rats when compared with those in the MCD group. Some of the components in SWQGT were reported to modulate metabolism and exert anti-obesity effects, for example, Quercetin prevents high-fat diet-induced obesity in mice, and its anti-obesity effects may be related to the regulation of lipogenesis (Jung et al., 2013). However, serum indicators yield very limited insights in the MCD-diet model (Ibrahim et al., 2016), and whether SWQGT could improve metabolic parameters needs further verification using other models based on the intake of high fat diets.

Network pharmacology is considered to be a powerful tool in investigating the complex mechanisms of multi-components TCM formulae (Zuo et al., 2018). In our network pharmacology approach, 53 putative targets from 26 components included TNF-α, IL-1β, etc, and were related to toll-like receptor signaling pathway, TNF signaling pathway, cytokine-cytokine receptor interaction, and NF-κB signaling pathway, which indicated that SWQGT may regulated inflammation response. MAPK signaling pathway and PI3K-AKT signaling pathway were also involved in the predicted mechanisms of SWQGT against NAFLD/NASH. Further experiments were carried out to explore the underlying mechanisms of SWQGT based on the results from enriched pathways.

The transcription factor NF-κB plays a vital role in regulating inflammatory response (Liu et al., 2017). Studies have shown that the inhibition of IKK/NF-κB signaling can reduce liver inflammation and steatosis effectively (Wang et al., 2014). The signal-regulated kinase (ERK) 1/2, a member of the mitogen-activated protein kinases (MAPKs), is activated by the upstream MAPK/ERK kinase (MEK) 1/2 signals (Kim and Choi, 2015). Then ERK1/2 can activate NF-κB by stimulating IKK, causing the release of inflammatory factors, such as TNF-α and IL-1β, and promoting the development of NASH (Lee et al., 2017; Cai et al., 2018). Inhibiting the ERK/NF-κB pathway is an effective way to reduce liver inflammation and hepatocyte apoptosis (Yu et al., 2019). We showed in this study that SWQGT down-regulated the levels of the ERK/NF-κB pathway-related proteins in the liver tissues of MCD diet-fed rats significantly, and decreased the protein levels of TNF-α, IL-1β, and IL-6. The pro-inflammatory gene MCP-1 was also lowered by SWQGT treatment. These cytokines are important factors in promoting hepatic steatosis and inflammation, causing apoptosis of liver cells (Zhang et al., 2016; Henkel et al., 2018). In accordance with these results, increased MPO activity and mRNA expression of EMR1, which are indicators of neutrophil and macrophage accumulation, respectively, were also alleviated by SWQGT treatment. These results demonstrate that SWQGT is able to modulate NF-κB pathway and reduce liver inflammation. During an inflammatory response, other signals from p38 MAPK, AKT, and TLR4 can also activate the NF-κB pathway (Lee et al., 2017). Per our results, SWQGT down-regulated p38 MAPK signaling but not AKT and TLR4 in MCD diet-fed rats. So we speculate that SWQGT might regulate NF-κB through ERK1/2 and p38 MAPK signals in MCD diet-fed rats.

The mammalian target of rapamycin (mTOR) can realize changes in external signals, such as growth factors and energy, and exert its regulatory effects on cell growth and proliferation (Kim et al., 2013). The above signals derived from ERK1/2 and Akt can also activate mTOR by inhibiting the tuberous sclerosis complex 2 (Mendoza et al., 2011). Here, we observed the activation of ERK1/2 and mTOR but not AKT in MCD diet-fed rats. The regulation of Akt phosphorylation in the livers of MCD diet-fed animals seems controversial in many studies. The levels of Akt phosphorylation have been reported to be unregulated, down regulated, and unaltered in previous studies (Lee et al., 2013; Li et al., 2016; Zheng et al., 2018; Lee et al., 2019). The differences might be caused by species, strain, sex and composition of gut microbiota, as well as dietary fat content (Van Herck et al., 2017). Hence, we speculate that the activation of mTOR in MCD diet-fed rats is associated with the activation of the ERK1/2 but not the AKT signal pathway.

The activation of mTOR can inhibit LC3I/LC3II conversion *via* the suppression of the UNC-51-like autophagy activating kinase (ULK) 1 (Wang et al., 2016). Autophagy is a lysosomal-dependent degradation process that functions to disassemble unnecessary or dysfunctional components, and maintain cell homeostasis (Beau et al., 2011). Studies have found that autophagy dysfunction has a close association with inflammation, and paves the way for the development of NAFLD/NASH (Fukuo et al., 2014). Contrarily, enhancing autophagy can help degrade hepatic lipids by lippophagy and reduce lipid accumulation (Czaja, 2016). The process will further suppress NF-κB and lessen inflammation (Trocoli and Djavaheri-Mergny, 2011). Activating autophagy has been reported to improve NASH in animal experiments effectively (Chen et al., 2019). In the current research, SWQGT inhibited mTOR activation, increased p-ULK1 and LC3II, and reduced p62 expression in MCD diet-fed rats, which suggests that SWQGT could promote autophagy flux by inhibiting mTOR.

Lastly, there are several limitations in our study. First, although SWQGT improved major indicators of NASH in the livers, its effect on metabolic parameters remains unclear due to the limitation of the MCD diet model. Mild weight loss was seen after high-dose of SWQGT treatment. Second, no improvement in ALT and AST levels was seen when SWQGT did alleviate inflammation and steatosis in the livers. Third, bioactive ingredients and their predicted targets used to construct the ingredient-target network may be influenced by the abundance and accuracy of public databases. Finally, SWQGT is multi-herbal formula, and the active ingredients are unknown. Identification of quality markers based on biological effects and their application in quality control will be important for the consistency of efficacy between batches.

## Conclusions

**Figure 8:**
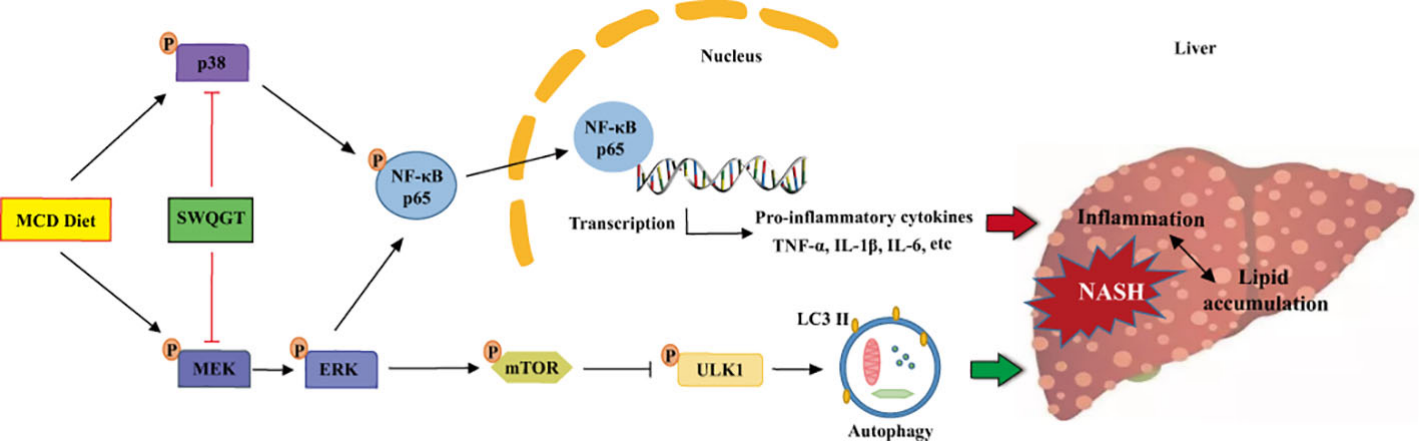
The schematic diagram of SWQGT modulating NF-κB signal pathway and autophagy against MCD diet-induced NASH.

In conclusion, SWQGT improved NASH in MCD diet-fed rats, and its protective effect was manifested specifically in reducing hepatomegaly, reducing liver lipid accumulation, and improving inflammation. What is more, this effect could be linked to its down-regulation of NF-κB through ERK1/2 and p38 MAPK signals and activation of autophagy by mTOR inhibition (**Figure 8**).

## Data Availability Statement

All datasets generated for this study are included in the article/**Supplementary Material**.

## Ethics Statement

The animal study was reviewed and approved by the Institutional Animal Care and Use Committee of the Shenzhen People's Hospital. The same committee approved this research (Approval Document No. LL-KT-201701017).

## Author Contributions

XZ and SP developed the concept of this study. LX, SL, LG, SQ, HW, SW, JF, XZ, and SP designed and performed experiments. LX and SL analyzed data. LX and SL prepared the draft and final version of the manuscript. All authors read and approved the final manuscript.

## Funding

This work was supported by grants from National Natural Science Foundation of China (81503221, 81703939, 81903914, 81903760), Shenzhen Fundamental Research and Discipline Layout Project (JCYJ20170307095556333, JCYJ20170413093108233, JCYJ20160427183814675, JCYJ20170307100726777), Natural Science Foundation of Guangdong Province (2017A030313659, 2014A030310365), and China Postdoctoral Science Foundation (2019M653300, 2018M633290).

## Conflict of Interest

The authors declare that the research was conducted in the absence of any commercial or financial relationships that could be construed as a potential conflict of interest.

## References

[B1] Allaire M. Rautou P. E. Codogno P. Lotersztajn S. (2019). Autophagy in liver diseases: Time for translation? J. Hepatol. 70, 985–998. 10.1016/j.jhep.2019.01.026 30711404

[B2] Bagherniya M. Nobili V. Blesso C. N. Sahebkar A. (2018). Medicinal plants and bioactive natural compounds in the treatment of non-alcoholic fatty liver disease: A clinical review. Pharmacol. Res. 130, 213–240. 10.1016/j.phrs.2017.12.020 29287685

[B3] Beau I. Mehrpour M. Codogno P. (2011). Autophagosomes and human diseases. Int. J. Biochem. Cell Biol. 43, 460–464. 10.1016/j.biocel.2011.01.006 21256243

[B4] Bedossa P. (2018). Diagnosis of non-alcoholic fatty liver disease/non-alcoholic steatohepatitis: Why liver biopsy is essential. Liv. Int. 38 1, 64–66. 10.1111/liv.13653 29427497

[B5] Bessone F. Razori M. V. Roma M. G. (2019). Molecular pathways of nonalcoholic fatty liver disease development and progression. Cell Mol. Life Sci. 76, 99–128. 10.1007/s00018-018-2947-0 30343320 PMC11105781

[B6] Botros M. Sikaris K. A. (2013). The de ritis ratio: the test of time. Clin. Biochem. Rev. 34, 117–130. 24353357 PMC3866949

[B7] Cai J. Zhang X. J. Li H. (2018). Role of innate immune signaling in non-alcoholic fatty liver disease. Trends Endocrinol. Metab. 29, 712–722. 10.1016/j.tem.2018.08.003 30131212

[B8] Cai F. F. Bian Y. Q. Wu R. Sun Y. Chen X. L. Yang M. D. (2019). Yinchenhao decoction suppresses rat liver fibrosis involved in an apoptosis regulation mechanism based on network pharmacology and transcriptomic analysis. Biomed. Pharmacother. 114, 108863. 10.1016/j.biopha.2019.108863 30991286

[B9] Chen R. Wang Q. Song S. Liu F. He B. Gao X. (2016). Protective role of autophagy in methionine-choline deficient diet-induced advanced nonalcoholic steatohepatitis in mice. Eur. J. Pharmacol. 770, 126–133. 10.1016/j.ejphar.2015.11.012 26593434

[B10] Chen X. Chan H. Zhang L. Liu X. Ho I. H. T. Zhang X. (2019). The phytochemical polydatin ameliorates non-alcoholic steatohepatitis by restoring lysosomal function and autophagic flux. J. Cell Mol. Med. 00, 1–11. 10.1111/jcmm.14320 PMC6533566 30973211

[B11] Czaja M. J. (2016). Function of autophagy in nonalcoholic fatty liver disease. Dig. Dis. Sci. 61, 1304–1313. 10.1007/s10620-015-4025-x 26725058 PMC4838507

[B12] Fang H. Y. Zeng H. W. Lin L. M. Chen X. Shen X. N. Fu P. (2017). A network-based method for mechanistic investigation of Shexiang Baoxin Pill's treatment of cardiovascular diseases. Sci. Rep. 7, 43632. 10.1038/srep43632 28272527 PMC5341564

[B13] Fazel Y. Koenig A. B. Sayiner M. Goodman Z. D. Younossi Z. M. (2016). Epidemiology and natural history of non-alcoholic fatty liver disease. Metabol. 65, 1017–1025. 10.1016/j.metabol.2016.01.012 26997539

[B14] Filozof C. Goldstein B. J. Williams R. N. Sanyal A. (2015). Non-Alcoholic Steatohepatitis: Limited Available Treatment Options but Promising Drugs in Development and Recent Progress Towards a Regulatory Approval Pathway. Drugs 75, 1373–1392. 10.1007/s40265-015-0437-3 26201461 PMC4532706

[B15] Fiorucci S. Biagioli M. Distrutti E. (2018). Future trends in the treatment of non-alcoholic steatohepatitis. Pharmacol. Res. 134, 289–298. 10.1016/j.phrs.2018.07.014 30021122

[B16] Fukuo Y. Yamashina S. Sonoue H. Arakawa A. Nakadera E. Aoyama T. (2014). Abnormality of autophagic function and cathepsin expression in the liver from patients with non-alcoholic fatty liver disease. Hepatol. Res. 44, 1026–1036. 10.1111/hepr.12282 24299564

[B17] Garattini S. Grignaschi G. (2017). Animal testing is still the best way to find new treatments for patients. Eur. J. Intern. Med. 39, 32–35. 10.1016/j.ejim.2016.11.013 27916437

[B18] Hamosh A. Scott A. F. Amberger J. S. Bocchini C. A. Mckusick V. A. (2005). Online Mendelian Inheritance in Man (OMIM), a knowledgebase of human genes and genetic disorders. Nucleic Acids Res. 33, 514–517. 10.1093/nar/gki033 PMC539987 15608251

[B19] He B. Wu L. Xie W. Shao Y. Jiang J. Zhao Z. (2017). The imbalance of Th17/Treg cells is involved in the progression of nonalcoholic fatty liver disease in mice. BMC Immunol. 18, 33. 10.1186/s12865-017-0215-y 28646856 PMC5483270

[B20] Henkel J. Coleman C. D. Schraplau A. Johrens K. Weiss T. S. Jonas W. (2018). Augmented liver inflammation in a microsomal prostaglandin E synthase 1 (mPGES-1)-deficient diet-induced mouse NASH model. Sci. Rep. 8, 16127. 10.1038/s41598-018-34633-y 30382148 PMC6208405

[B21] Ibrahim S. H. Hirsova P. Malhi H. Gores G. J. (2016). Animal Models of Nonalcoholic Steatohepatitis: Eat, Delete, and Inflame. Dig. Dis. Sci. 61, 1325–1336. 10.1007/s10620-015-3977-1 26626909 PMC4838538

[B22] Jadeja R. Devkar R. V. Nammi S. (2014). Herbal medicines for the treatment of nonalcoholic steatohepatitis: current scenario and future prospects. Evid. Based. Complement. Alternat. Med. 2014, 648308. 10.1155/2014/648308 24987431 PMC4060323

[B23] Jung C. H. Cho I. Ahn J. Jeon T. I. Ha T. Y. (2013). Quercetin Reduces High-Fat Diet-Induced Fat Accumulation in the Liver by Regulating Lipid Metabolism Genes. Phytother. Res. 27, 139–143. 10.1002/ptr.4687 22447684

[B24] Kim E. K. Choi E. J. (2015). Compromised MAPK signaling in human diseases: an update. Arch. Toxicol. 89, 867–882. 10.1007/s00204-015-1472-2 25690731

[B25] Kim K. H. Lee M. S. (2018). Pathogenesis of Nonalcoholic Steatohepatitis and Hormone-Based Therapeutic Approaches. Front. Endocrinol. (Lausanne) 9, 485. 10.3389/fendo.2018.00485 30197624 PMC6117414

[B26] Kim S. G. Buel G. R. Blenis J. (2013). Nutrient regulation of the mTOR complex 1 signaling pathway. Mol. Cells 35, 463–473. 10.1007/s10059-013-0138-2 23694989 PMC3887879

[B27] Kochi T. Shimizu M. Terakura D. Baba A. Ohno T. Kubota M. (2014). Non-alcoholic steatohepatitis and preneoplastic lesions develop in the liver of obese and hypertensive rats: suppressing effects of EGCG on the development of liver lesions. Cancer Lett. 342, 60–69. 10.1016/j.canlet.2013.08.031 23981577

[B28] Konerman M. A. Jones J. C. Harrison S. A. (2018). Pharmacotherapy for NASH: Current and emerging. J. Hepatol. 68, 362–375. 10.1016/j.jhep.2017.10.015 29122694

[B29] Kubes P. Mehal W. Z. (2012). Sterile inflammation in the liver. Gastroenterol. 143, 1158–1172. 10.1053/j.gastro.2012.09.008 22982943

[B30] Lau J. K. C. Zhang X. Yu J. (2018). Animal Models of Non-alcoholic Fatty Liver Diseases and Its Associated Liver Cancer. Adv. Exp. Med. Biol. 1061, 139–147. 10.1007/978-981-10-8684-7_11 29956212

[B31] Lee K. C. Chan C. C. Yang Y. Y. Hsieh Y. C. Huang Y. H. Lin H. C. (2013). Aliskiren attenuates steatohepatitis and increases turnover of hepatic fat in mice fed with a methionine and choline deficient diet. PloS One 8, e77817. 10.1371/journal.pone.0077817 24204981 PMC3804600

[B32] Lee J. W. Kim Y. I. Kim Y. Choi M. Min S. Joo Y. H. (2017). Grape seed proanthocyanidin inhibits inflammatory responses in hepatic stellate cells by modulating the MAPK, Akt and NF-kappaB signaling pathways. Int. J. Mol. Med. 40, 226–234. 10.3892/ijmm.2017.2997 28534957

[B33] Lee K. C. Hsieh Y. C. Chan C. C. Sun H. J. Huang Y. H. Hou M. C. (2019). Human relaxin-2 attenuates hepatic steatosis and fibrosis in mice with non-alcoholic fatty liver disease. Lab. Invest. 99, 1203–1216. 10.1038/s41374-019-0240-y 30918325

[B34] Lee S. (2015). Systems Biology - A Pivotal Research Methodology for Understanding the Mechanisms of Traditional Medicine. J. Pharmacopunct. 18, 11–18. 10.3831/KPI.2015.18.020 PMC4573803 26388998

[B35] Li W. Ma F. Zhang L. Huang Y. Li X. Zhang A. (2016). S-Propargyl-cysteine Exerts a Novel Protective Effect on Methionine and Choline Deficient Diet-Induced Fatty Liver via Akt/Nrf2/HO-1 Pathway. Oxid. Med. Cell Longev. 2016, 4690857. 10.1155/2016/4690857 27313828 PMC4893438

[B36] Li H. Toth E. Cherrington N. J. (2018). Asking the Right Questions With Animal Models: Methionine- and Choline-Deficient Model in Predicting Adverse Drug Reactions in Human NASH. Toxicol. Sci. 161, 23–33. 10.1093/toxsci/kfx253 29145614 PMC6454421

[B37] Lin S. C. Heba E. Bettencourt R. Lin G. Y. Valasek M. A. Lunde O. (2017). Assessment of treatment response in non-alcoholic steatohepatitis using advanced magnetic resonance imaging. Aliment. Pharmacol. Ther. 45, 844–854. 10.1111/apt.13951 28116801 PMC5346270

[B38] Liu T. Zhang L. Joo D. Sun S. C. (2017). NF-kappaB signaling in inflammation. Signal. Transduction Targ. Ther. 2, 17023. 10.1038/sigtrans.2017.23 PMC5661633 29158945

[B39] Marcolin E. Forgiarini L. F. Rodrigues G. Tieppo J. Borghetti G. S. Bassani V. L. (2013). Quercetin decreases liver damage in mice with non-alcoholic steatohepatitis. Basic Clin. Pharmacol. Toxicol. 112, 385–391. 10.1111/bcpt.12049 23331460

[B40] Mendoza M. C. Er E. E. Blenis J. (2011). The Ras-ERK and PI3K-mTOR pathways: cross-talk and compensation. Trends Biochem. Sci. 36, 320–328. 10.1016/j.tibs.2011.03.006 21531565 PMC3112285

[B41] Pierantonelli I. Svegliati-Baroni G. (2019). Nonalcoholic Fatty Liver Disease: Basic Pathogenetic Mechanisms in the Progression From NAFLD to NASH. Transpl. 103, e1–e13. 10.1097/TP.0000000000002480 30300287

[B42] Pinero J. Bravo A. Queralt-Rosinach N. Gutierrez-Sacristan A. Deu-Pons J. Centeno E. (2017). DisGeNET: a comprehensive platform integrating information on human disease-associated genes and variants. Nucleic Acids Res. 45, D833–D839. 10.1093/nar/gkw943 27924018 PMC5210640

[B43] Ru J. Li P. Wang J. Zhou W. Li B. Huang C. (2014). TCMSP: a database of systems pharmacology for drug discovery from herbal medicines. J. Cheminform. 6, 13. 10.1186/1758-2946-6-13 24735618 PMC4001360

[B44] Tahan V. Eren F. Avsar E. Yavuz D. Yuksel M. Emekli E. (2007). Rosiglitazone attenuates liver inflammation in a rat model of nonalcoholic steatohepatitis. Dig. Dis. Sci. 52, 3465–3472. 10.1007/s10620-007-9756-x 17436085

[B45] Tanaka N. Ichijo T. Okiyama W. Mutou H. Misawa N. Matsumoto A. (2006). Laparoscopic findings in patients with nonalcoholic steatohepatitis. Liv. Int. 26, 32–38. 10.1111/j.1478-3231.2005.01198.x 16420508

[B46] Trocoli A. Djavaheri-Mergny M. (2011). The complex interplay between autophagy and NF-κB signaling pathways in cancer cells. Am. J. Canc. Res. 1, 629. PMC3189824 21994903

[B47] Ullah R. Rauf N. Nabi G. Ullah H. Shen Y. Zhou Y. D. (2019). Role of Nutrition in the Pathogenesis and Prevention of Non-alcoholic Fatty Liver Disease: Recent Updates. Int. J. Biol. Sci. 15, 265–276. 10.7150/ijbs.30121 30745819 PMC6367556

[B48] Van Herck M. A. Vonghia L. Francque S. M. (2017). Animal Models of Nonalcoholic Fatty Liver Disease-A Starter's Guide. Nutrients 9, E1072. 10.3390/nu9101072 28953222 PMC5691689

[B49] Wang X. A. Zhang R. She Z. G. Zhang X. F. Jiang D. S. Wang T. (2014). Interferon regulatory factor 3 constrains IKKbeta/NF-kappaB signaling to alleviate hepatic steatosis and insulin resistance. Hepatol. 59, 870–885. 10.1002/hep.26751 24123166

[B50] Wang Y. Nie H. Zhao X. Qin Y. Gong X. (2016). Bicyclol induces cell cycle arrest and autophagy in HepG2 human hepatocellular carcinoma cells through the PI3K/AKT and Ras/Raf/MEK/ERK pathways. BMC Canc. 16, 742. 10.1186/s12885-016-2767-2 PMC5031284 27654866

[B51] Watanabe T. Jono H. Han J. Lim D. J. Li J. D. (2004). Synergistic activation of NF-κB by nontypeable Haemophilus influenzae and tumor necrosis factor α. Proc. Natl. Acad. Sci. U. S. A. 101, 3563–3568. 10.1073/pnas.0400557101 14993593 PMC373502

[B52] Xu X. Zhang W. X. Huang C. Li Y. Yu H. Wang Y. H. (2012). A Novel Chemometric Method for the Prediction of Human Oral Bioavailability. Int. J. Mol. Sci. 13, 6964–6982. 10.3390/ijms13066964 22837674 PMC3397506

[B53] Younossi Z. M. Koenig A. B. Abdelatif D. Fazel Y. Henry L. Wymer M. (2016). Global epidemiology of nonalcoholic fatty liver disease-Meta-analytic assessment of prevalence, incidence, and outcomes. Hepatol. 64, 73–84. 10.1002/hep.28431 26707365

[B54] Yu Q. Wu L. Liu T. Li S. Feng J. Mao Y. (2019). Protective effects of levo-tetrahydropalmatine on hepatic ischemia/reperfusion injury are mediated by inhibition of the ERK/NF-kappaB pathway. Int. Immunopharmacol. 70, 435–445. 10.1016/j.intimp.2019.02.024 30856394

[B55] Zhang S. J. Chen Z. X. Jiang K. P. Cheng Y. H. Gu Y. L. (2008). The effect of QuYuHuaTanTongLuo Decoction on the non-alcoholic steatohepatitis. Complement. Ther. Med. 16, 192–198. 10.1016/j.ctim.2007.08.004 18638709

[B56] Zhang X. Han J. Man K. Li X. Du J. Chu E. S. (2016). CXC chemokine receptor 3 promotes steatohepatitis in mice through mediating inflammatory cytokines, macrophages and autophagy. J. Hepatol. 64, 160–170. 10.1016/j.jhep.2015.09.005 26394162

[B57] Zheng Y. Wang M. Zheng P. Tang X. Ji G. (2018). Systems pharmacology-based exploration reveals mechanisms of anti-steatotic effects of Jiang Zhi Granule on non-alcoholic fatty liver disease. Sci. Rep. 8, 13681. 10.1038/s41598-018-31708-8 30209324 PMC6135841

[B58] Zuo H. Zhang Q. Su S. Chen Q. Yang F. Hu Y. (2018). A network pharmacology-based approach to analyse potential targets of traditional herbal formulas: An example of Yu Ping Feng decoction. Sci. Rep. 8, 11418. 10.1038/s41598-018-29764-1 30061691 PMC6065326

