# Supplementary material for: Si-Wei-Qing-Gan-Tang Improves Non-Alcoholic Steatohepatitis by Modulating the Nuclear Factor-κB Signal Pathway and Autophagy in Methionine and Choline Deficient Diet-Fed Rats
Source: Front Pharmacol. 2020 May 1;11:530. doi: 10.3389/fphar.2020.00530 (PMC7206618; doi:10.3389/fphar.2020.00530)
Supplement: Supplementary file 1 [file DataSheet_1.docx]

Supplementary Material

**Supplement Figure S1.** UPLC chromatograms of SWQGT and its single herb.

**
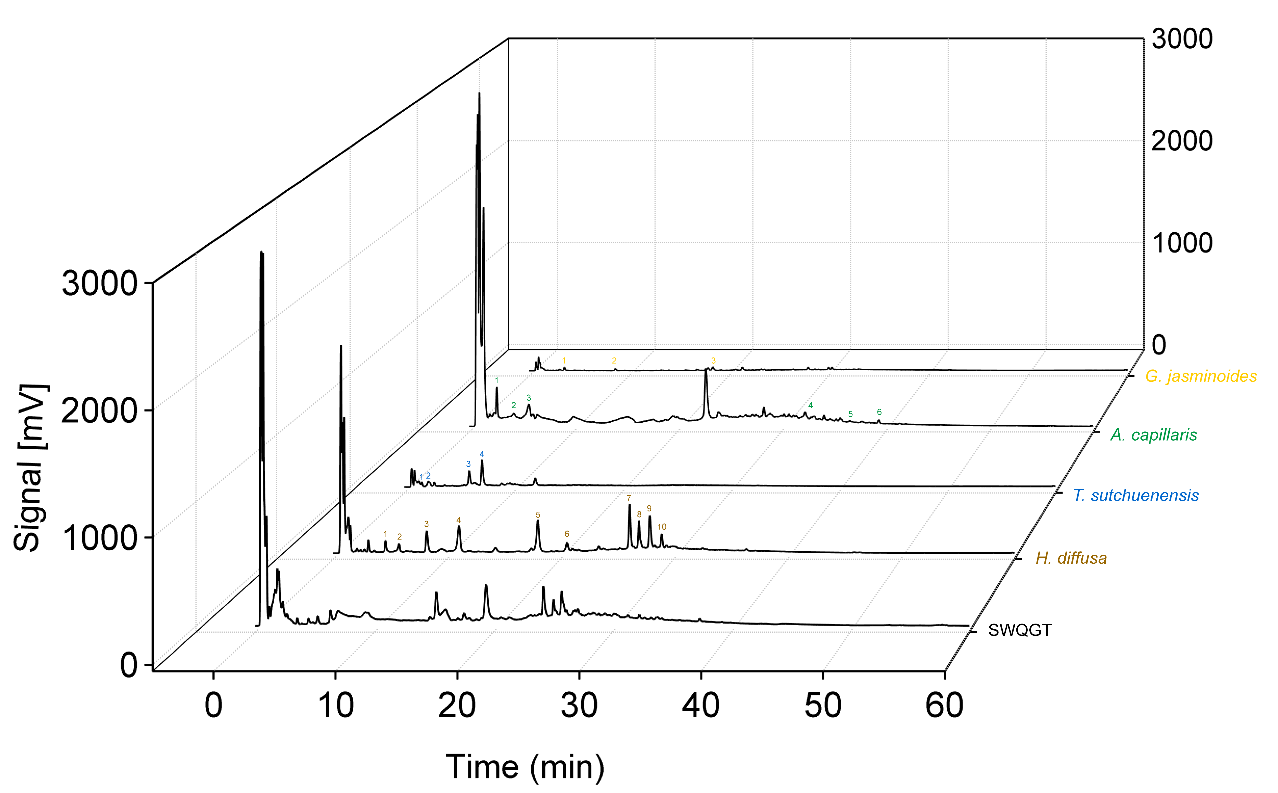
**

**Supplement Table S1.** Putative Identification of Compounds Using Mass Spectrometry in the herbs of SWQGT

| **Peak** | **T_R_ (time)** | **MS^-^ (m/z)** | **Tentative identification** | **Reference** |
| --- | --- | --- | --- | --- |
| H1 | 4.7 | 241 | n-Pentadecanoic acid | [1], [2] |
| H2 | 5.8 | 431 | Asperuloside acid | [1], [3] |
| H3 | 8.3 | 459 | Wogonin-O-glu acid | [1], [4] |
| H4 | 11.1 | 119 | 4-Vinyl phenol | [1], [2] |
| H5 | 18.1 | 625 | Quercetin-3-O-(2-O-glucopyranosyl)-β-D-glucopyranside | [1], [5] |
| H6 | 20.3 | 595 | Quercetin-3-O-sambubioside | [1], [5] |
| H7 | 26.1 | 831 | Quercetin-3-O-[2-O-(6-O-E-sinapoyl)-β-D-glucopyranosyl]-β-D-glucopyanoside | [1], [5] |
| H8 | 27.0 | 801 | Quercetin-3-O-[2-O-(6-O-E-feruloyl)-β-D-glucopyranosyl]-β-D-glucopyanoside | [1], [5], [6] |
| H9 | 27.9 | 549 | (Z)-6-O-p-Coumaroyl scandoside methyl ester | [1], [7] |
| H10 | 29.0 | 549 | (E)-6-O-p-Coumaroyl scandoside methyl ester | [1], [5], [6] |
| T1 | 1.6 | 301 | Quercetin | [8], [9] |
| T2 | 2.5 | 575 | Daucosterol | [9] |
| T3 | 6.1 | 577 | Kaempferol-3,7-bisrhamnoside | [10] |
| T4 | 7.2 | 289 | Catechin | [11] |
| A1 | 1.4 | 387 | (+)-Medioresinol | [12] |
| A2 | 4.4 | 137 | Salicylic acid | [12] |
| A3 | 5.9 | 161 | Umbelliferone | [12] |
| A4 | 32.8 | 285 | Demethoxycapillarisin | [13] |
| A5 | 36.7 | 327 | Luteolin-3’,4’,7-trimethyl ether | [13] |
| A6 | 39.5 | 329 | Eupalitin | [14] |
| G1 | 3.6 | 241 | 6β-Hydroxygenipin | [15] |
| G2 | 8.7 | 477 | Jasminoside N | [16] |
| G3 | 18.5 | 581 | Lyoniresinol-9-O-β-D-glucopyranoside | [17] |

**Reference**

[1] Chen, R., He, J., Tong, X., Tang, L., Liu, M. (2016). The *Hedyotis diffusa* Willd. (Rubiaceae): A Review on Phytochemistry, Pharmacology, Quality Control and Pharmacokinetics. *Molecules* 21, E710.

[2] Liu, Z.G., Luo, J.B., Chen, F.L. (2005). The Pilot Study of Volatile Compounds in *Hedyotis diffusa* from different sources. *Tradit. Chin. Drug Res. Clin. Pharm.* 16, 132-134.

[3] Nishihama, Y., Masuda, K., Yamaki, M., Takagi, S., Sakina, K. (1981). Three new iridoid glucosides from *Hedyotis diffusa*. *DlantaMedica* 43, 28-33.

[4] Li, C.M., Zhao, Y.Y., Guo, Z.M., Zhang, X.L., Xue, X.Y., Liang, X.M. (2014). Effective 2D-RPLC/RPLC enrichment and separation of micro-components from *Hedyotis diffusa* Willd and characterization by using ultra-performance liquid chromatography/quadrupole time-of-flight mass spectrometry. *J. Pharm. Biomed. Anal.* 99, 35-44.

[5] Liu, E.H., Zhou, T., Li, G.B., Li, J., Huang, X.N., Pan, F., et al. (2012). Characterization and identification of iridoid glucosides, flavonoids and anthraquinones in *Hedyotis diffusa* by high-performance liquid chromatography/electrospray ionization tandem mass spectrometry. *J. Sep. Sci.* 35, 263-272.

[6] Li, D.X., Schmitz, O.J. (2015). Comprehensive two-dimensional liquid chromatography tandem diode array detector (DAD) and accurate mass QTOF-MS for the analysis of flavonoids and iridoid glycosides in *Hedyotis diffusa*. *Anal. Bioanal. Chem.* 407, 231-240.

[7] Ji, B.Y., Fan, C.Q., Fei, L.X., Ma, Y. (2014). Advance on the chemical and pharmacological effects studies of *Hedyotis diffusa*. *Chin. J. Exp. Tradit. Med. Form*. 20, 235-240.

[8] Li, M.R., Li, L.Q., Ma, X.Y., Ha, X.B. (1986). Content determination of quercetin in *Taxillus chinensis*, *Taxillus Sutchuenensis* and *Scurrulaparasitica*. *West China J. Pharm. Sci.* 1, 131-134.

[9] Chen, J.T, Feng, F. (2007). Studies on chemical constituents of *Taxillus sutchuenenisis*. *Chin. Med. Mat.* 30, 1393-1395.

[10] Yang, L., Lin, J., Zhou, B., Liu, Y., Zhu, B. (2017). Activity of compounds from *Taxillus sutchuenensis* as inhibitors of HCV NS3 serine protease. *Nat. Prod. Res.* 31, 487-491.

[11] Li, M.R., Li, L.Q., Li, P. (1987). Studies on the Fiavonoids of *Taxillus sutchuenensis* (Lecomte) Danser and *T. sutchuenensis* var. duclouxii (Lecomte) Kiuined. *West China J. Pharm. Sci.* 12, 738-740.

[12] Zhao, Y., Geng, C.A., Sun, C.L., Ma, Y.B., Huang, X.Y., Cao, T.W. et al. (2014). Polyacetylenes and anti-hepatitis B virus active constituents from *Artemisia capillaris*. *Fitoterapia* 95, 187-193.

[13] Wu, T.S., Tsang, Z.J., Wu, P.L., Lin, F.W., Li, C.Y., Teng, C.M., et al. (2001). New Constituents and Antiplatelet Aggregation and Anti-HIV Principles of *Artemisia capillaris*. *Bioorg. Med. Chem.* 9, 77-83.

[14] Tang, H.Q., Hu, J., Yang, L., Tan, R.X. (2000). Terpenoids and flavonoids from *Artemisia* species. *Planta Med*. 66, 391-393.

[15] Peng, K., Yang, L., Zhao, S., Chen, L., Zhao, F., Qiu, F. (2013). Chemical constituents from the fruit of *Gardenia jasminoides* and their inhibitory effects on nitric oxide production. *Bioorg. Med. Chem. Lett.* 23, 1127-1131.

[16] Yu, Y., Gao, H., Dai, Y., Wang, Y., Chen, H.R., Yao, X.S. (2010). Monoterpenoids from the Fruit of *Gardenia jasminoides*. *Helv. Chim. Acta* 93, 763-771.

[17] Yu, Y., Gao, H., Dai, Y., Li, X.X., Li, J.M., Yao, X.S. (2010). A new lignan from *Gardenia jasminoides*. *Chin. Tradit. Herbal Drugs* 41, 509-514.

**Supplementary Table S2.** 26 components from the 4 herbs and their candidate targets

| **Number** | **Component** | **OB%** | **DL** | **Herb** | **Targets** |
| --- | --- | --- | --- | --- | --- |
| 1 | Skrofulein | 30.35 | 0.3 | *Artemisia capillaris* Thunb | COX1, COX2, DPP4 |
| 2 | Beta-sitosterol | 36.91 | 0.75 | *Artemisia capillaris* Thunb, *Gardenia jasminoides* Ellis, *Hedyotis diffusa* Willd | ADRA1A, ADRA1B, ADRB2, BAX, BCL2, CASP3, CASP8, CASP9, CHRM1, CHRM2, CHRM3, CHRM4, CHRNA2, CHRNA7, COX1, COX2, DRD1, GABRA1, GABRA2, GABRA3, GABRA5, HSP90AA1, HSP90AB1, HTR2A, JUN, KCNH2, MAP2, NCOA2, OPRM1, PDE3A, PGR, PIK3CG, PON1, PRKCA, SCN5A, SLC6A4, TGFB1 |
| 3 | Genkwanin | 37.13 | 0.24 | *Artemisia capillaris* Thunb | AR, CALM1, CALM2, CALM3, CHEK1, COX1, COX2, DPP4, ESR2, HSP90AA1, HSP90AB1, NCOA1, NCOA2, NOS2, PRKCA, PRSS1, RXRA |
| 4 | Eupatolitin | 42.55 | 0.37 | *Artemisia capillaris* Thunb | AR, CDK2, COX2, DPP4, ESR2, HSP90AA1, HSP90AB1, NOS2, PRSS1, SCN5A, TOP2A, TOP2B |
| 5 | Eupalitin | 46.11 | 0.33 | *Artemisia capillaris* Thunb | AR, CALM1, CALM2, CALM3, CDK2, CHEK1, COX1, COX2, DPP4, ESR2, F2, GSK3B, HSP90AA1, HSP90AB1, MAPK14, NOS2, PRKCA, PRSS1, SCN5A |
| 6 | Quercetin | 46.43 | 0.28 | *Artemisia capillaris* Thunb, *Gardenia jasminoides* Ellis, *Hedyotis diffusa* Willd, *Taxillus sutchuenensis (Lecomte)* Danser | ABCG2, ACACA, ACHE, ACPP, ADRB2, AHR, AHSA1, AKR1B1, AKT1, ALOX5, AR, BAX, BCL2, BCL2L1, BIRC5, CASP3, CASP8, CASP9, CAV1, CCL2, CCNB1, CCND1, CD40LG, CDK1, CDK2, CHEK2, CHUK, CLDN4, COL1A1, COL3A1, COX1, COX2, CRP, CTSD, CXCL10, CXCL11, CXCL2, CYP1A1, CYP1A2, CYP1B1, CYP3A4, DCAF5, DIO1, DPP4, DUOX2, E2F1, E2F2, EGF, EGFR, EIF6, ELK1, ERBB2, ERBB3, F10, F2, F3, F7, FOS, GABRA1, GJA1, GSTM1, GSTM2, GSTP1, HAS2, HERC5, HIF1A, HK2, HMOX1, HSF1, HSP90AA1, HSP90AB1, HSPA5, HSPB1, ICAM1, IFNG, IGF2, IGFBP3, IL10, IL1A, IL1B, IL2, IL6, IL8, INSR, IRF1, JUN, KCNH2, MAOB, MAPK1, MGAM, MMP1, MMP2, MMP3, MMP9, MPO, Myc, NCF1, NCOA2, NFE2L2, NFKBIA, NKX3-1, NOS3, NPEPPS, NQO1, NR1I2, NR1I3, ODC1, PARP1, PCOLCE, PIK3CG, PLAT, PLAU, PON1, POR, PPARA, PPARD, PPARG, PRKCA, PRKCB, PRSS1, PSMD3, PTEN, PTGER3, RAF1, RASA1, RASSF1, RB1, RELA, RUNX1T1, RUNX2, RXRA, SCN5A, SELE, SERPINE1, SLC2A4, SOD1, SPP1, STAT1, SULT1E1, TGFB1, THBD, TNFA, TNFB, TNFC, TNFSF13, TOP1, TOP2A, TOP2B, TP53, VCAM1, VEGF, XDH |
| 7 | Areapillin | 48.96 | 0.41 | *Artemisia capillaris* Thunb | AR, CALM1, CALM2, CALM3, COX2, DPP4, ESR2, F10, F2, F7, HSP90AA1, HSP90AB1, NCOA1, NCOA2, NOS2, PRSS1, PTPN1, SCN5A, TOP2A, TOP2B |
| 8 | Isorhamnetin | 49.6 | 0.31 | *Artemisia capillaris* Thunb | ACHE, AKR1B1, AR, CALM1, CALM2, CALM3, CCNA2, CDK2, CHEK1, COX1, COX2, DPP4, ESR1, ESR2, F2, F7, GABRA1, GRIA2, GSK3B, HSP90AA1, HSP90AB1, MAOB, MAPK14, NCF1, NCOA1, NCOA2, NOS2, NOS3, OLR1, PIK3CG, PIM1, PPARD, PPARG, PRKCA, PRSS1, PTPN1, PYGM, RELA, XDH |
| 9 | Demethoxycapillarisin | 52.33 | 0.25 | *Artemisia capillaris* Thunb | AKR1B1, CDK2, COX1, COX2, DPP4, ESR1, GSK3B, HSP90AA1, HSP90AB1, MAPK14, PIK3CG, PPARG, PRKCA, PTPN1 |
| 10 | Isoarcapillin | 57.4 | 0.41 | *Artemisia capillaris* Thunb | AR, CALM1, CALM2, CALM3, COX2, DPP4, ESR2, F10, F2, F7, HSP90AA1, HSP90AB1, NCOA2, NOS2, PRSS1, PTPN1, SCN5A, TOP2A, TOP2B |
| 11 | Capillarisin | 57.56 | 0.31 | *Artemisia capillaris* Thunb | AKR1B1, CALM1, CALM2, CALM3, CDK2, CHEK1, COX1, COX2, DPP4, GSK3B, HSP90AA1, HSP90AB1, MAPK14, MMP9, PIK3CG, PRKCA, PTPN1, PYGM |
| 12 | Artepillin A | 68.32 | 0.24 | *Artemisia capillaris* Thunb | ADRA1A, ADRB2, AR, CDK2, CHRM1, COX1, COX2, DPP4, ESR1, ESR2, F2, GSK3B, NOS2, NOS3, PDE3A, PRSS1 |
| 13 | 4'-Methylcapillarisin | 72.18 | 0.35 | *Artemisia capillaris* Thunb | CALM1, CALM2, CALM3, CDK2, COX2, DPP4, F2, GSK3B, HSP90AA1, HSP90AB1, MAPK14, PRKCA, PTPN1, SCN5A |
| 14 | Crocetin | 35.3 | 0.26 | *Gardenia jasminoides* Ellis | ADRA1A, ADRA1B, CHRM1, CHRM2, CHRM3, COX2, GABRA1, GABRA2, GABRA3, GABRA5, NCOA2, VCAM1 |
| 15 | Ammidin | 34.55 | 0.22 | *Gardenia jasminoides* Ellis | CHRM1, COX2, DPP4, F2, GABRA1, MAOB, PIK3CG, PRKCA |
| 16 | Sudan III | 84.07 | 0.59 | *Gardenia jasminoides* Ellis | CCNA2, CDK2, COX2, DPP4, ESR1, ESR2, F2, F7, GSK3B, MAPK10, MAPK14, PIM1, PRKCA |
| 17 | Kaempferol | 41.88 | 0.24 | *Gardenia jasminoides* Ellis | ACHE, ADRA1B, AHR, AHSA1, AKR1C3, AKT1, ALOX5, AR, BAX, BCL2, CALM1, CALM2, CALM3, CASP3, CDK1, CHRM1, CHRM2, COX1, COX2, CYP1A1, CYP1A2, CYP1B1, CYP3A4, DIO1, DPP4, F2, F7, GABRA1, GABRA2, GSTM1, GSTM2, GSTP1, HAS2, HMOX1, HSP90AA1, HSP90AB1, ICAM1, IKBKB, INSR, JUN, MAPK8, MMP1, NCOA2, NOS2, NOS3, NR1I2, NR1I3, PGR, PIK3CG, PPARG, PPP3CA, PRKCA, PRSS1, PSMD3, RELA, SELE, SLC2A4, SLC6A2, SLPI, STAT1, TNFA, TNFB, TNFC, TNFSF13, TOP2A, TOP2B, VCAM1, XDH |
| 18 | Stigmasterol | 43.83 | 0.76 | *Gardenia jasminoides* Ellis, *Hedyotis diffusa* Willd | ADH1C, ADRA1A, ADRA1B, ADRA2A, ADRB1, ADRB2, AKR1B1, CHRM1, CHRM2, CHRM3, CHRNA7, COX1, COX2, CTRB1, CTRB2, GABRA1, GABRA3, HTR2A, LTA4H, MAOA, MAOB, NCOA1, NCOA2, NR3C2, PGR, PLAU, PRKCA, RXRA, SCN5A, SLC6A2, SLC6A3 |
| 19 | Mandenol | 42 | 0.19 | *Gardenia jasminoides* Ellis | COX1, COX2, NCOA2 |
| 20 | Isoimperatorin | 45.46 | 0.23 | *Gardenia jasminoides* Ellis | COX2 |
| 21 | Ethyl oleate (NF) | 32.4 | 0.19 | *Gardenia jasminoides* Ellis | NCOA2 |
| 22 | 5-hydroxy-7-methoxy-2-(3,4,5-trimethoxyphenyl)chromone | 51.96 | 0.41 | *Gardenia jasminoides* Ellis | ADRB2, AR, BACE1, BACE2, CACNA2D1, CALM1, CALM2, CALM3, CHEK1, COX1, COX2, DPP4, ESR1, ESR2, F10, F2, GSK3B, HSP90AA1, HSP90AB1, KCNH2, KCNMA1, MAPK14, NCOA1, NCOA2, NOS2, NOS3, PPARG, PRSS1, SCN5A, TOP2A, TOP2B |
| 23 | 3-Methylkempferol | 60.16 | 0.26 | *Gardenia jasminoides* Ellis | AR, CDK2, COX1, COX2, DPP4, GSK3B, HSP90AA1, HSP90AB1, MAPK14, NOS2, PIK3CG, PRKCA |
| 24 | 2-methoxy-3-methyl-9,10-anthraquinone | 37.83 | 0.21 | *Hedyotis diffusa* Willd | ADRA1A, ADRA1B, ADRA1D, ADRB2, CHRM1, CHRM2, CHRM3, CHRM4, CHRM5, CHRNA7, COX1, COX2, DRD1, ESR1, GABRA1, HRH1, HSP90AA1, HSP90AB1, HTR2A, HTR2C, NCOA2, OPRD1, OPRM1, PDE3A, PIK3CG, PKIA, PRKCA, RXRA, SCN5A, SLC6A2, SLC6A3, SLC6A4 |
| 25 | Poriferasterol | 43.83 | 0.76 | *Hedyotis diffusa* Willd | NR3C2, PGR |
| 26 | Sitosterol | 36.91 | 0.75 | *Taxillus sutchuenensis (Lecomte)* Danser | NCOA2, NR3C2, PGR |
